# Boron Prevents Oral Acrylamide Damage in Bone Marrow and Hematologic Parameters of Wistar Rats

**DOI:** 10.1007/s12011-025-04610-4

**Published:** 2025-04-07

**Authors:** Faysal Selimoğlu, Mustafa Cengiz, Canan Vejselova Sezer, Adnan Ayhanci, Fatma Gür, Özge Yıldırım, Bahri Gür, Ahmet Musmul

**Affiliations:** 1 Department of Biotechnology, Faculty of Science, Necmettin Erbakan University, Konya, Türkiye; 2 Department of Elementary Education, Faculty of Education, Siirt University, Siirt, Türkiye; 3 Department of Biology, Faculty of Science and Arts, Kütahya Dumlupınar University, Kütahya, Türkiye; 4 Department of Biology, Faculty of Science, Eskisehir Technical University, Eskişehir, Türkiye; 5 Department of Biology, Faculty of Science, Eskişehir Osmangazi University, Eskişehir, Türkiye; 6 Department of Dentistry Services, Atatürk University, Erzurum, Türkiye; 7 Department of Biochemistry, Faculty of Science, Iğdır University, Iğdır, Türkiye; 8 Department of Medical Services and Techniques, Faculty of Medicine, Eskişehir Osmangazi University, Eskişehir, Türkiye

**Keywords:** Acrylamide, Myelotoxicity, Boron, Hematologic toxicity, Rats

## Abstract

The purpose of this study is to measure the effects of acrylamide (AA) on bone marrow and blood and the protective efficacy of boric acid (B) in rats. The animals were divided into five groups: control, B, low-dose B + AA, and high-dose B + AA. After the 15 th day of the experiment, their blood and bone marrow were harvested to be kept for hematological analyses. While white blood cells (WBC) increased following the administration of AA, bodyweight, red blood cells, platelets, hemoglobin, hematocrit, and bone marrow nucleated cell counts significantly decreased in number. Conversely, bodyweight and all these hematological parameters significantly increased apart from WBC in the B + AA groups AA when compared to the control. However, high-dose B was more effective than low-dose B in preventing AA-induced bone marrow and hematological damage. The present study suggests that B treatment could help protect bone marrow and hematological parameters against AA-induced toxicity.

**Graphical Abstract:**

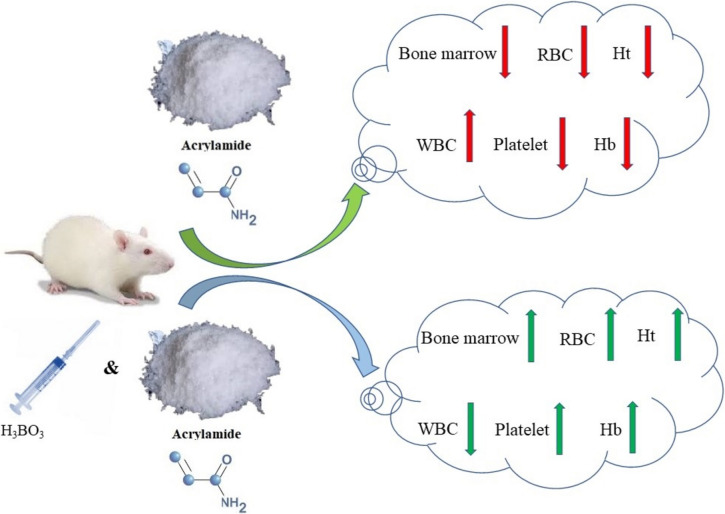

## Highlights

• Acrylamide caused bone marrow toxicity.

• Boron has a protective effect against acrylamide-induced toxicity

• Acrylamide-induced reduction in body weight was prevented by boron.

• Acrylamide-induced white blood cell increase was reduced by boron supplementation.

## Introduction

Acrylamide (AA), C_3_H_5_NO, is a colorless and odorless chemical compound that is widely utilized in industrial areas for a variety of functions including wastewater treatment, mining, textile, paper production, surface coating, and cosmetics manufacture. AA is commonly used in clinics and research laboratories to prepare polyacrylamide gels [1]. It develops in foods heated over 120 °C, such as chips, French fries, cereal, and coffee. Furthermore, the higher the temperature, the greater the concentration. It also possesses neurotoxic, genotoxic, hepatotoxic, nephrotoxic, hematological toxicity, and carcinogenic effects [2]. The International Agency for Research on Cancer classifies AA as a Group 2 A chemical in 112,124. Substances in Group 2 A have been identified as potentially carcinogenic to humans. Despite various research demonstrating that acrylamide causes various forms of cancer in animals, its carcinogenicity in humans remains unknown. However, this should not mean that it poses no risks for humans considering its genotoxicity, mutagenicity, and clastogenicity [3]. In creating the intermedia substance, that is glycidamide, AA is metabolized in the liver by CYP2E1 enzymes. Glycidamide is claimed to be approximately one thousand times more mutagenic and genotoxic than the AA itself. After oral ingestion, inhalation, or dermal exposure, AA accumulates in the blood at higher levels than it does in other tissues. Furthermore, it causes deterioration of hematological parameters, reduces erythrocyte membrane resistance, and delays Hb synthesis or degradation [4, 5].

Some in vivo and in vitro investigations have revealed that trace elements with antioxidant characteristics suppress the beginning and progression of carcinogenesis, preventing cell death and change. Furthermore, they have suggested that certain trace elements can significantly reduce cellular toxicities caused by cytotoxic agents [6]. Boron is a trace element that exhibits anti-lipid peroxidative, anti-oxidant [7, 8] activity, boosts the therapeutic efficacy of anti-neoblastic drugs, and diminishes the toxic side effects of cytotoxic treatments including cisplatin and cyclophosphamide [9, 10]. While there are known to be studies that have explored AA-induced blood toxicity in the literature [11], the effects of this substance upon the bone marrow remain to be clarified. Therefore, our study is the first of its kind in the sense that it investigates the effects of acrylamide on the bone marrow. All these taken into account, the present study aims to evaluate the protective effects of boron given to experimental rats in different dosages upon AA-induced bone marrow and hematological toxicity damage in rats.

## Material and Methods

### Chemicals

Pure boric acid (99%), boron compounds, and AA are commercially available (Chempure). For this study, B and AA, the dosages of which had been adjusted to those published in the literature, were orally administered (forced oral administration) to the experimental rats and suspended in distilled water [12, 13]. The experimental study was approved by the Local Ethics Committee for Animal Experiments at Eskisehir Osmangazi University (No: 151/8062020). All of the animals were purchased from Kobay Incorporated Company Experimental Animal Production Laboratory of the Ministry of Health of the Republic of Turkey. The rats were kept under standard conditions (humidity 45–50%, temperature 22 ± 2 °C, light 12 h/dark 12 h) and fed a standard pellet diet and drinking water.

### Treatment Protocols

**Fig. 1:**
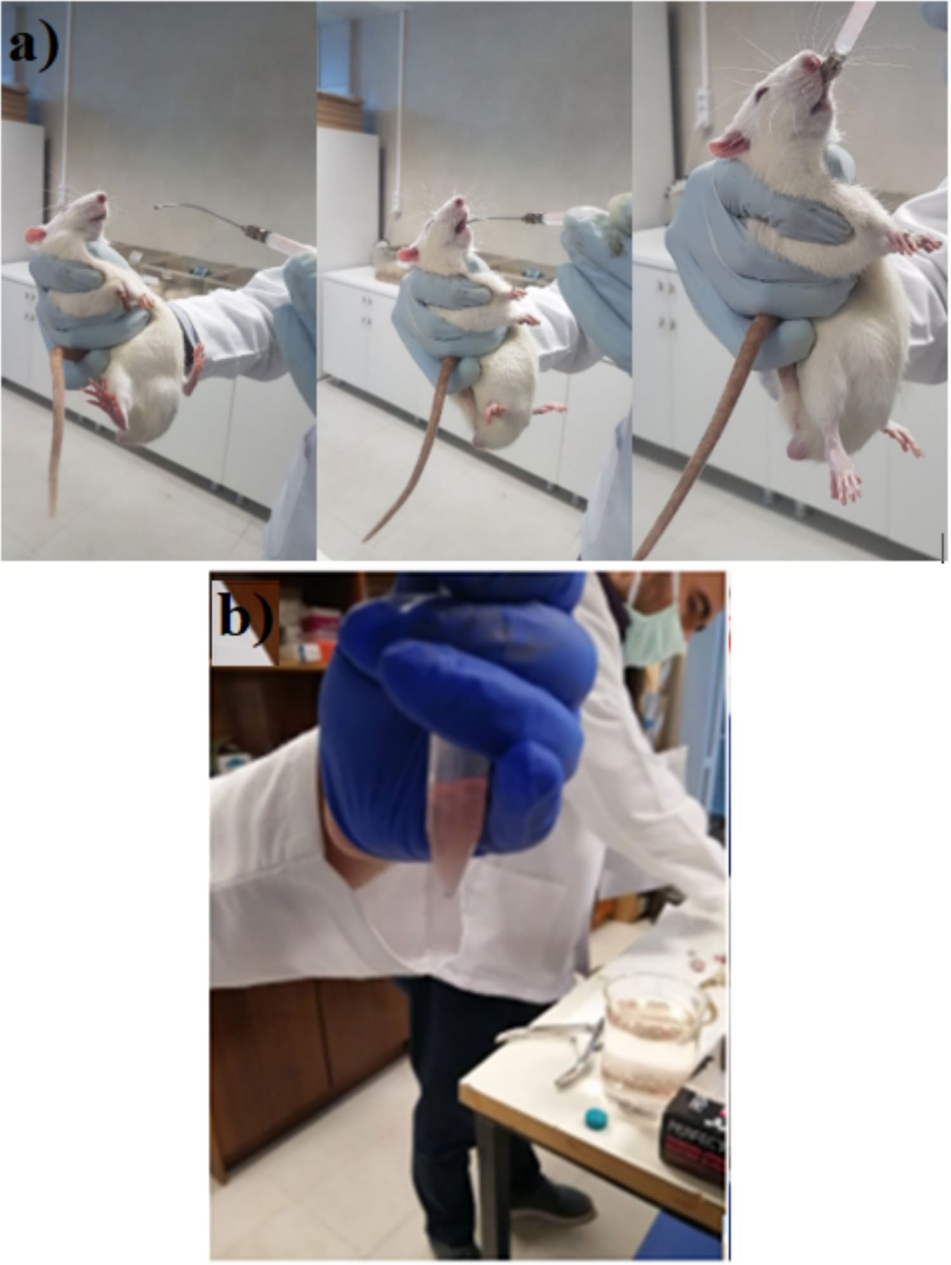
**a** Gavage application. **b** The sample homogenization

Wistar male rats of 200–250 gr were selected for the experiments and were randomly divided into five groups of six members: Control (1), AA (2), B (3), 10 B + AA (4), and 20 B + AA (5). In this study, the animals were given low-dose (10 mg/kg) and high-dose (20 mg/kg) boric acid (B) intraperitoneal (i.p.) for 14 days because these dosages have been shown not to have any toxic effects [12]. Because the literature indicates that the 10 mg/kg B dose is not toxic, no separate group was developed to which this dosage would be applied to prevent animal waste. Throughout the last 10 days of the experiment, AA (38.27 mg/kg) gavage was applied to the rats (Fig. 1a). Their blood and bone marrow samples were collected via cardiac puncture under anesthesia on the 15 th day, when the experiment ended and the animals were all sacrificed.

1. Control group (Group 1): 0.5 mL saline for 14 days
2. AA group (Group 2): 38.27 mg/kg gavage
3. B group (Group 3): 20 mg/kg i.p. for 14 days
4. B + AA group (Group 4): 10 mg/kg boric acid + acrylamide
5. B + AA group (Group 5): 20 mg/kg boric acid + acrylamide

### Isolation and Counting of Bone Marrow Nucleated Cells

A femoral muscle was resected in the rats slaughtered under ketamine/xylazine anesthesia [14]. By cutting both its ends, the femur itself was resected. The bone marrow was then collected in a graduated test tube after 5 mL of pressured saline was injected from the top of the femur. By continuously sucking and emptying the liquid contents with the same syringe, the samples were homogenized without producing bubbles (Fig. 1b). The test tube was centrifuged at 3000 rpm for 5 min, and all but the final 0.5 mL of supernatant were removed. By pipetting the pellet and supernatant (0.5 mL) using a Pasteur pipette, the pellet and supernatant were homogenized once more. Finally, the samples were counted on a blood count device [15].

### Measurement of Hematological Parameters

Hematological parameters were determined by taking blood from the heart. The blood samples were placed in EDTA tubes, and RBC, WBC, Hb, PLT, and Ht levels were measured using an automated blood analyzer [14, 16].

### Statistical Analyses

The experimental data obtained from this study were evaluated using the program package version of “SPSS 18.0 for Windows.” Results of the peripheral blood cells (leucocytes, thrombocytes, and erythrocytes) were analyzed via blood serum, hemoglobin hematocrit, and bone marrow cell counts; the differences between the groups were evaluated by “Kruskal–Wallis One Way Analysis of Variance on Ranks. Median (25–75%).” The values achieving *p* < 0.001 for the experimental groups were considered statistically significant.

## Results

### Bodyweight

**Fig. 2:**
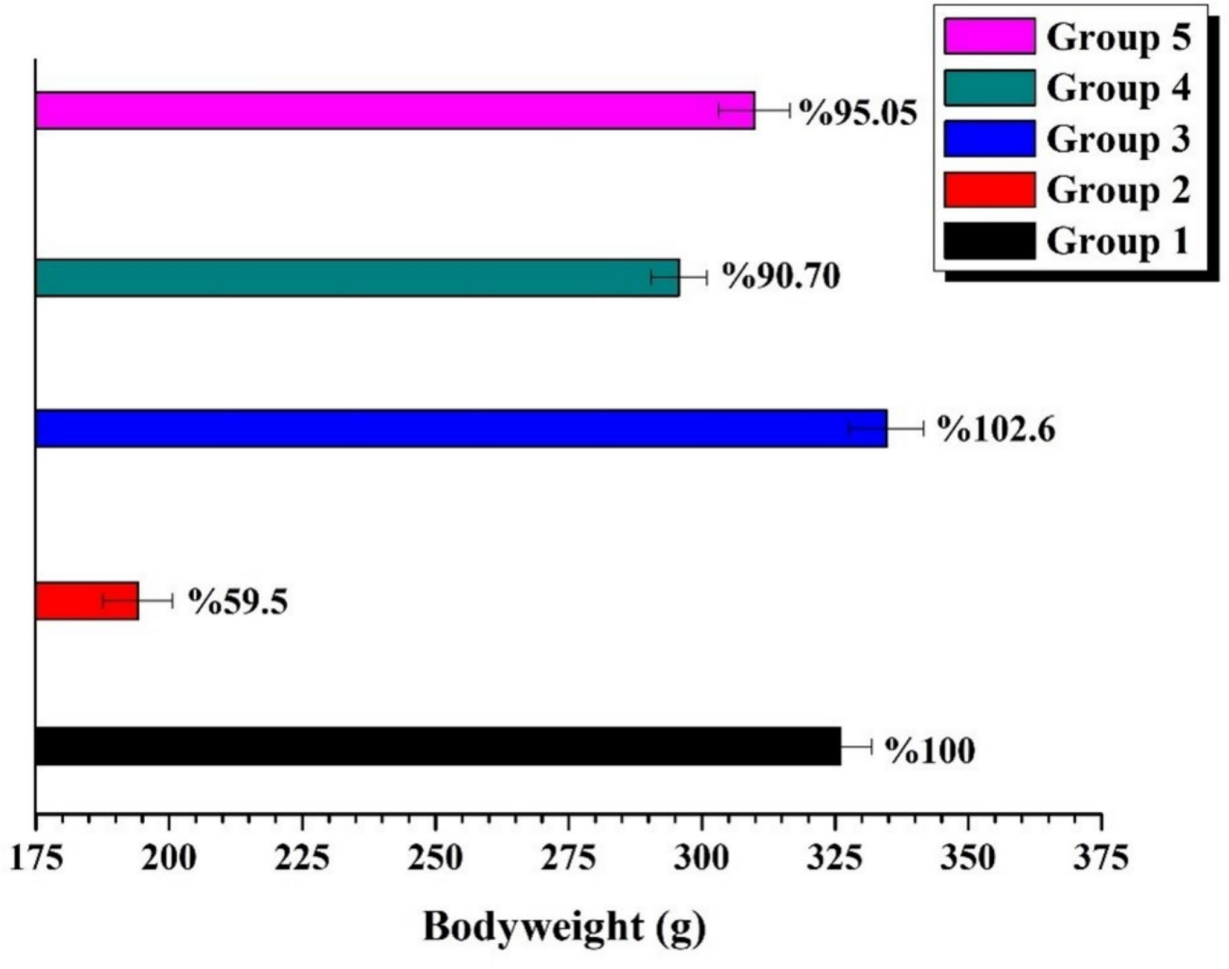
Bodyweight of the rats

The rats in all experimental groups were weighed before the injection and sacrifice procedures (Fig. 2).

The weight of the animals in the experimental group receiving AA had fallen considerably as compared to the Control Group. The weight of the animals increased in the group that received only B. The animals in the B + AA groups, on the other hand, lost much more weight than the rats in the AA alone groups.

### Hematological Parameters

**Fig. 3:**
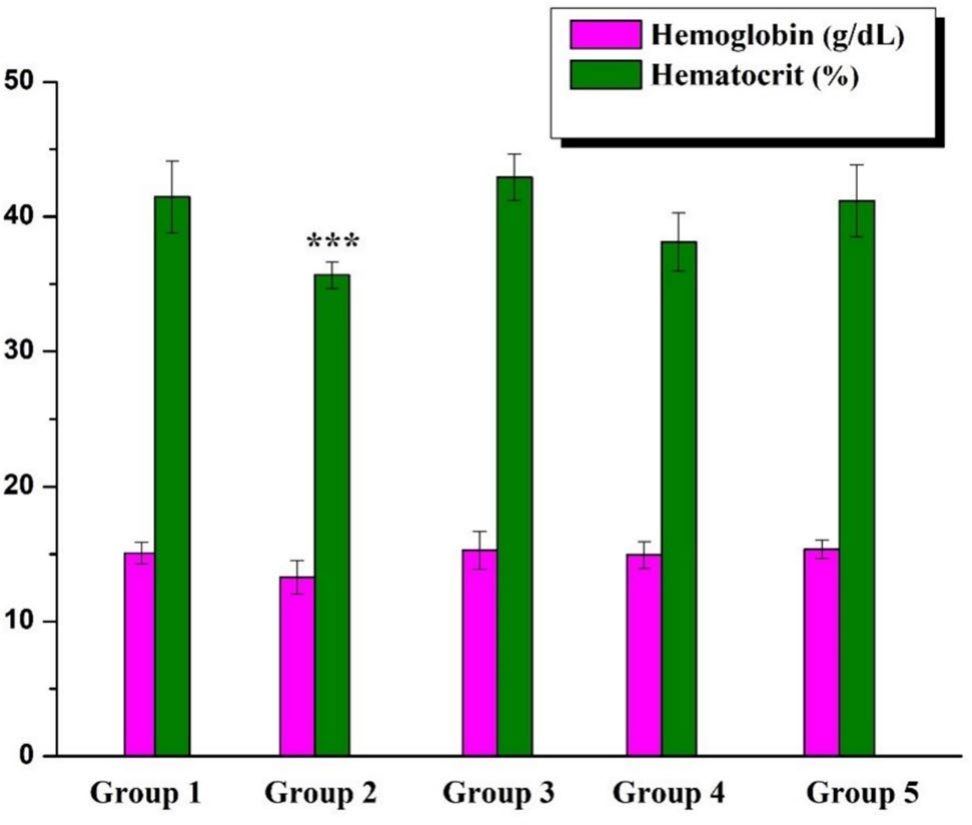
Hemoglobin and hematocrit levels (*p* < 0.001: *** stands for a remarkable difference when compared to the Control group)

**Fig. 4:**
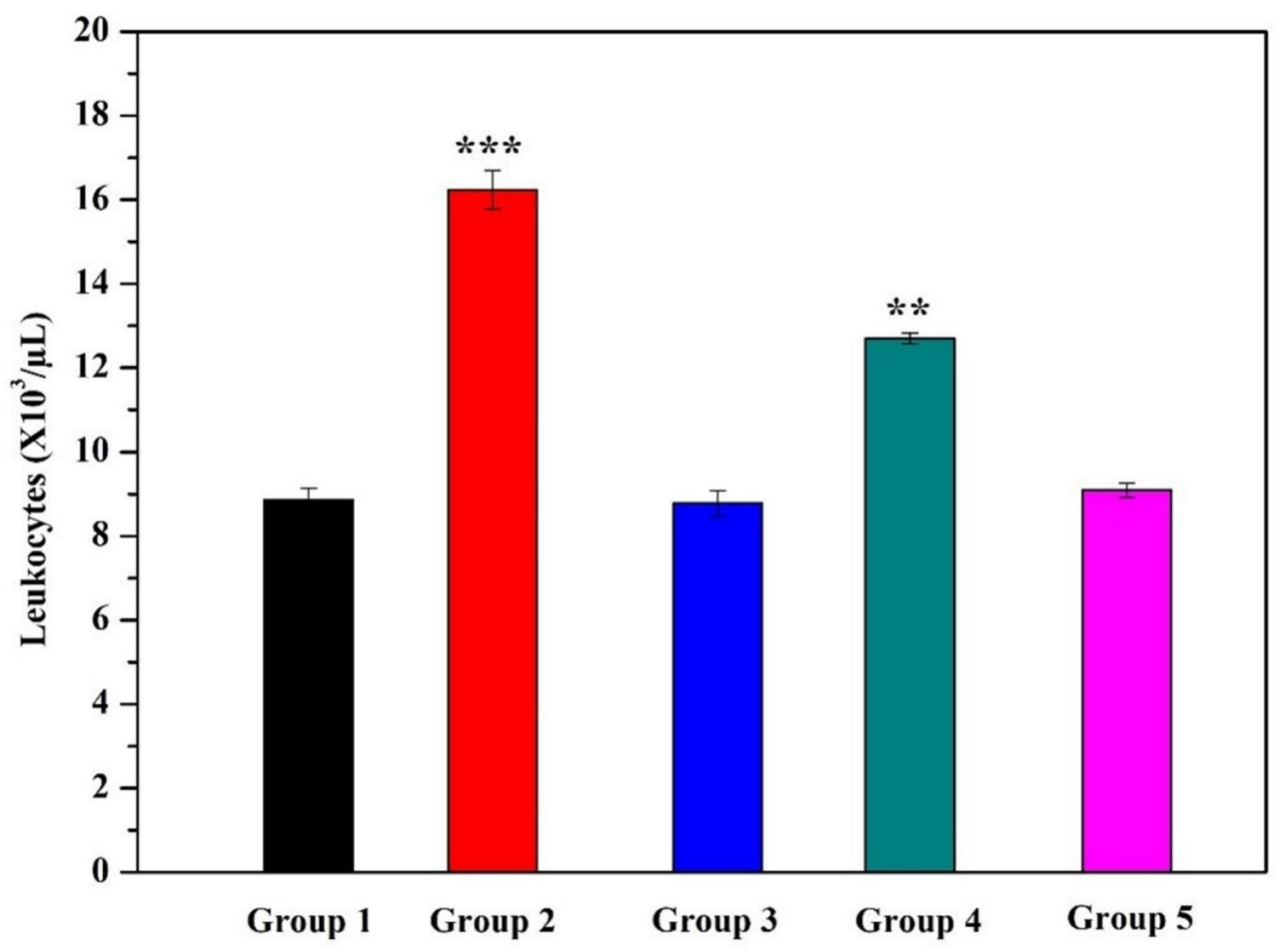
Leukocyte levels (*p* < 0.001: *** stands for a remarkable difference when compared to the Control group; ** stands for a difference when compared to the Control group)

**Fig. 5:**
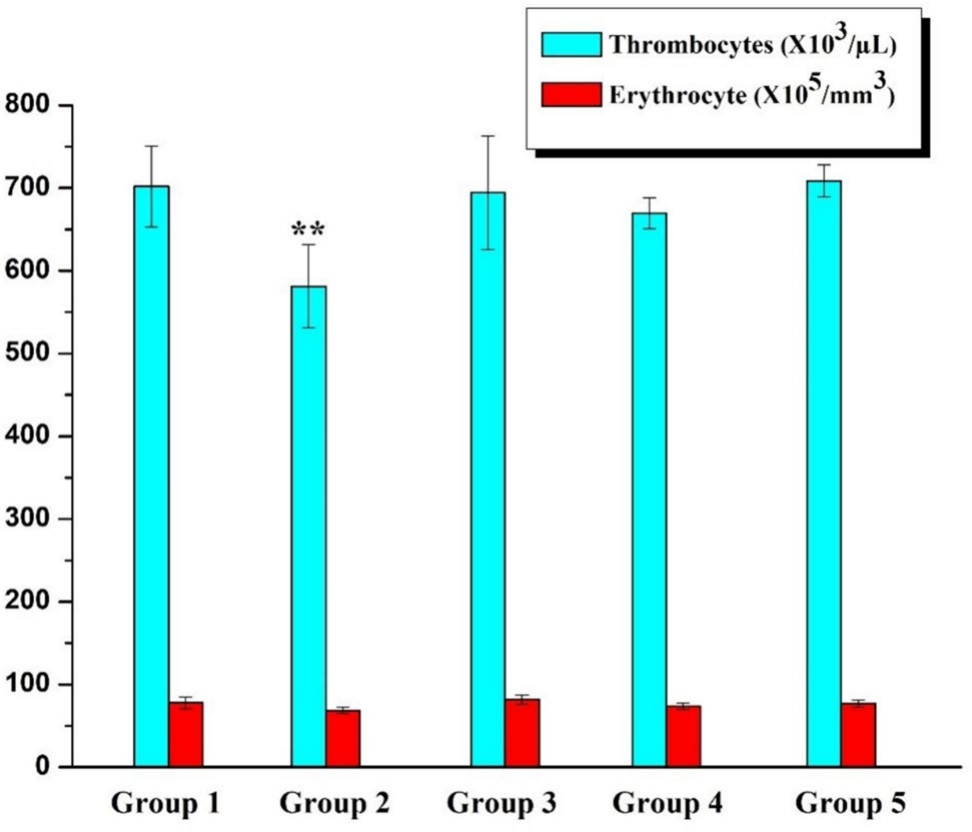
Thrombocyte and erythrocyte levels (*p* < 0.001: ** stands for a difference when compared to the Control group)

Ht, Hb levels, RBC, WBC, and PLT counts in rats receiving saline and B were comparable to those in the Control Group. Compared to the Control Group, AA-treated rats showed significant decreases in Ht, Hb levels, RBC, and PLT counts (*p* < 0.001), whereas WBC levels increased. The same parameters improved dramatically in rats receiving AA with B. Although statistically insignificant, 20 B can be considered slightly more effective than 10 B (Figs. 3, 4, and 5).

### Bone Marrow Cellularity Assessment

**Fig. 6:**
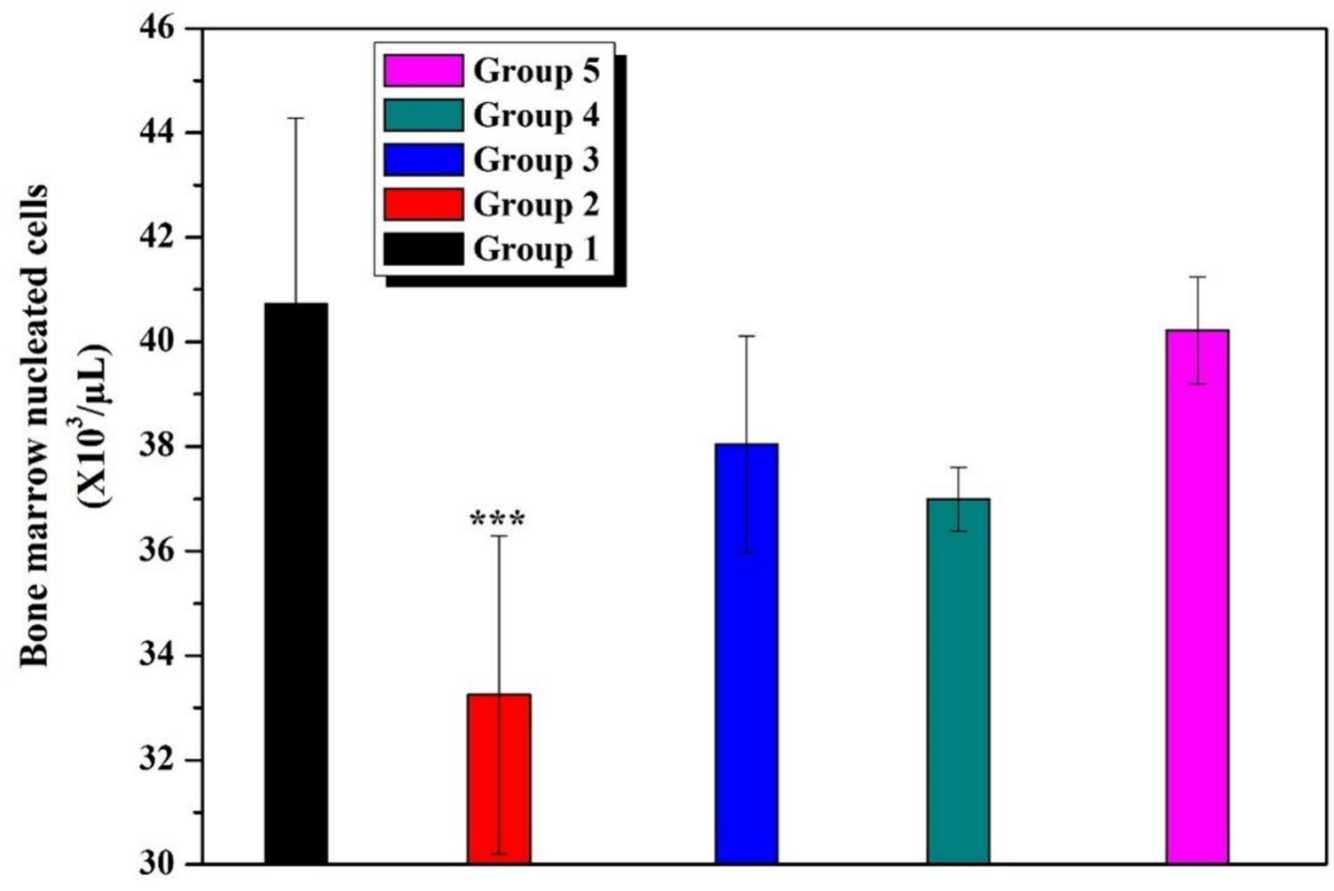
Bone marrow nucleated cells (*p* < 0.001: *** stands for a remarkable difference when compared to the Control group)

Bone marrow cellularity in the rats given saline and B was similar to that of the Control group. As for the bone marrow cellularity of AA-given rats, it was significantly reduced compared to that of the Control group (*p* < 0.001). However, as far as the bone marrow cellularity of the rats given AA along with B is concerned, it significantly improved. Although statistically insignificant, 20 B was found to have a slightly higher effect than did 10 B when expressed numerically (Fig. 6).

## Discussion

The purpose of this study is to determine whether or not boron exerts a protective effect upon AA-induced bone marrow and blood toxicity. In this study, in addition to the remarkable fall in Ht and Hb levels, significant decreases were also observed in RBC and PLT counts in AA-treated rats when compared to the Control group (*p* < 0.001). On the other hand, there was an increase in WBC level consistent with the results of studies in the literature [11, 17, 18]. Commonly known as leukocytes, white blood cells (WBCs) form a part of the immune system cells. The number of these cells was considerably greater in the rats treated in this study (*p* < 0.05) than those left untreated, thus indicating that AA had activated the immune system [11, 17]. Correspondingly, a previous study found that the number of RBC, Ht, Hb, and platelet (PLT) values decreased while the WBC count increased in the AA-treated group compared to the control group [18]. The number of bone marrow cells in the group administered AA, on the other hand, decreased significantly as compared to that of the Control group. Our study is the first of its kind in demonstrating the impact of AA upon the number of bone marrow cells. Several studies have revealed that antioxidant nutrients protect cells from the damaging effects of environmental contaminants [19].

Boron is a naturally occurring mineral that is widely utilized in industrial, agricultural, and cosmetic purposes besides its traditional use in health care. This trace element gets rapidly absorbed and disseminated throughout the body through passive diffusion once it has been delivered. Up to date, two different claims have been made that boron performs biochemical and physiological functions in both humans and animals. According to the first claim, B is not only involved in cell-membrane processes regulating hormone movement, but also in transmembrane signaling and regulating ion mobility [20, 21]. The second one claims that boron has is capable of operating as a metabolic regulator in several enzymatic systems [22]. Furthermore, boron plays a role in cellular membrane functions although its antioxidant mechanisms remain to be clarified [23]. In this study, boron was effective in preventing AA-induced hematotoxicity. This activity of boron is compatible with the literature data. For example, in one study, 200 mg/kg B increased the antioxidant defense system while lowering lipid peroxidation [24]. In another study, there was a 41% decrease in platelet count and a 96% decrease in leukocyte count in the experimental group given 200 mg/kg CP. However, as compared to the CP Group, there was a decrease in the CP + B Group in the leukocyte count by 50% and the platelet count by 20% [9].

According to our findings, the AA group had the lowest bodyweight averages. Similar to our findings, previous studies have also shown that AA to reduce the bodyweight. For instance, Donmez et al. [25] found that rats given 40 mg/kg AA lost up to 30% of their bodyweight while Li et al. [26] noted that the groups given 20 and 40 mg/kg AA suffered considerable weight loss. The fact that the 10 B + AA and 20 B + AA groups had higher bodyweight averages than the AA group alone could indicate that boron has a protective effect against acrylamide toxicity. There is no research in the literature demonstrating the potential effects of boron on AA-induced weight reduction.

Based on the data collected, it was determined that using B resulted in a considerable reduction in AA-induced bone marrow suppression. To conclude, boron is not toxic to bone marrow or blood cells on its own, but AA is toxic to the combination of bone marrow, leukocytes, and platelets. Boron is assumed to protect animals against the toxicity of AA, which has not been discussed AA, in the literature thus far. These findings suggest that, when given at proper doses, boron could be a potentially useful drug in the treatment of AA-induced damage, thus providing hope for the prevention and treatment of AA toxicity. Therefore, we are of the opinion that further studies are required to grasp the underlying mechanism of boron.

## Conclusions

Based on the data collected, it was determined that using B resulted in a considerable reduction in AA-induced bone marrow suppression. To conclude, boron is not toxic to bone marrow or blood cells on its own, but AA is toxic to the combination of bone marrow, leukocytes, and platelets. Boron is assumed to protect animals against the toxicity of AA, which has not been discussed AA, in the literature thus far. These findings suggest that, when given at proper doses, boron could be a potentially useful drug in the treatment of AA-induced damage, thus providing hope for the prevention and treatment of AA toxicity. Therefore, we are of the opinion that further studies are required to grasp the underlying mechanism of boron.

## Data Availability

No datasets were generated or analysed during the current study.

## References

[1] Acaroz U, Ince S, Arslan-Acaroz D, Gurler Z, Kucukkurt I, Demirel HH et al (2018) The ameliorative effects of boron against acrylamide-induced oxidative stress, inflammatory response, and metabolic changes in rats. Food Chem Toxicol 118:745–752. 10.1016/j.fct.2018.06.029 29913234 10.1016/j.fct.2018.06.029

[2] Gür F, Cengiz M, Gür B, Cengiz O, Sarıçiçek O, Ayhancı A (2023) Therapeutic role of boron on acrylamide-induced nephrotoxicity, cardiotoxicity, neurotoxicity, and testicular toxicity in rats: effects on Nrf2/Keap-1 signaling pathway and oxidative stress. J Trace Elem Med Biol 80:127274. 10.1016/j.jtemb.2023.127274 37562273 10.1016/j.jtemb.2023.127274

[3] Abdel-Daim MM, Abo El-Ela FI, Alshahrani FK, Bin-Jumah M, Al-Zharani M, Almutairi B et al (2020) Protective effects of thymoquinone against acrylamide-induced liver, kidney and brain oxidative damage in rats. Environ Sci Pollut Res 27:37709–37717. 10.1007/s11356-020-09516-3 10.1007/s11356-020-09516-3 32608003

[4] Arıhan O, Seringeç NB, Gürel EI, Dikmenoğlu NH (2011) Effects of oral acrylamide intake on blood viscosity parameters in rats. Clin Hemorheol Microcirc 47:45–52. 10.3233/CH-2010-1364 21321407 10.3233/CH-2010-1364

[5] Rawi SM, Marie M-AS, Fahmy SR, El-Abied SA (2012) Hazardous effects of acrylamide on immature male and female rats. African J Pharm Pharmacol. 6:1367–86. 10.5897/AJPP12.148

[6] Ağgül AG, Gür F, Gülaboğlu M (2021) Streptozotocin-induced oxidative stress in rats: the protective role of olive leaf extract. Bull Korean Chem Soc 42:180–187. 10.1002/bkcs.12157

[7] Cengiz M, Yildiz SC, Demir C, Şahin İK, Teksoy Ö, Ayhanci AJJoTEiM (2019) Biology. Hepato-preventive and anti-apoptotic role of boric acid against liver injury induced by cyclophosphamide. 53:1-7. 10.1016/j.jtemb.2019.01.013 10.1016/j.jtemb.2019.01.013 30910191

[8] Cengiz M, Baytar O, Şahin Ö, Kutlu HM, Ayhanci A, VejselovaSezer C, Gür B (2024) Biogenic synthesized bare and boron-doped copper oxide nanoparticles from Thymbra spicat ssp. spicata: in silico and in vitro studies. J Cluster Sci 35:265–84. 10.1007/s10876-023-02481-0

[9] Cengiz M (2018) Hematoprotective effect of boron on cyclophosphamide toxicity in rats. Cell Mol Biol (Noisy-le-grand) 64(5):62–65. 10.14715/cmb/2018.64.5.10 29729695

[10] Kan F, Kucukkurt I (2022) Investigation of the effect of boron on thyroid functions and biochemical parameters in hypothyroid induced-rats. J Biochem Mol Toxicol 36:e23186. 10.1002/jbt.23186 35924451 10.1002/jbt.23186

[11] BelhadjBenziane A, DilmiBouras A, Mezaini A, Belhadri A, Benali M (2019) Effect of oral exposure to acrylamide on biochemical and hematologic parameters in Wistar rats. Drug Chem Toxicol 42:157–166. 10.1080/01480545.2018.1450882 29589771 10.1080/01480545.2018.1450882

[12] Ince S, Kucukkurt I, Demirel HH, Acaroz DA, Akbel E, Cigerci IH (2014) Protective effects of boron on cyclophosphamide induced lipid peroxidation and genotoxicity in rats. Chemosphere 108:197–204. 10.1016/j.chemosphere.2014.01.038 24530163 10.1016/j.chemosphere.2014.01.038

[13] Uthra C, Shrivastava S, Jaswal A, Sinha N, Reshi MS, Shukla SJB (2017) Pharmacotherapy therapeutic potential of quercetin against acrylamide induced toxicity in rats. Biomed Pharmacother 86:705–714. 10.1016/j.biopha.2016.12.065 28039850 10.1016/j.biopha.2016.12.065

[14] Heinzel JC, Hercher D, Redl H (2020) The course of recovery of locomotor function over a 10-week observation period in a rat model of femoral nerve resection and autograft repair. Brain Behavior 10:e01580. 10.1002/brb3.1580 32097542 10.1002/brb3.1580 PMC7177579

[15] Ayhanci A, Uyar R, Aral E, Kabadere S, Appak S (2008) Protective effect of zinc on cyclophosphamide-induced hematoxicity and urotoxicity. Biol Trace Elem Res 126:186–193 18641924 10.1007/s12011-008-8189-5

[16] Ayhanci A, Heybeli N, Sahin İK, Cengiz M (2019) Myelosuppression and oxidative stress induced by cyclophosphamide in rats: the protective role of selenium. Adıyaman Univer J Sci 9:252–65. 10.37094/adyujsci.617016

[17] Ghorbel I, Chaabane M, Elwej A, Kallel C, GratiKamoun N, Najiba Z (2017) Extra Virgin olive oil mitigates hematotoxicity induced by acrylamide and oxidative damage in adult rats. Pharm Biomed Res 3:34–40. 10.18869/acadpub.pbr.3.1.34

[18] Raju J, Roberts J, Taylor M, Patry D, Chomyshyn E, Caldwell D et al (2015) Toxicological effects of short-term dietary acrylamide exposure in male F344 rats. Environ Toxicol Pharmacol 39:85–92. 10.1016/j.etap.2014.11.009 25473820 10.1016/j.etap.2014.11.009

[19] Hashem MM, Abo-El-Sooud K, Abd El-Hakim YM, Abdel-hamidBadr Y, El-Metwally AE, Bahy-El-Dien A (2022) The impact of long-term oral exposure to low doses of acrylamide on the hematological indicators, immune functions, and splenic tissue architecture in rats. Int Immunopharmacol 105:108568. 10.1016/j.intimp.2022.108568 35101847 10.1016/j.intimp.2022.108568

[20] Jabbar AAJ, Alamri ZZ, Abdulla MA, Salehen NA, Ibrahim IAA, Hassan RR et al (2024) Boric acid (boron) attenuates AOM-induced colorectal cancer in rats by augmentation of apoptotic and antioxidant mechanisms. Biol Trace Elem Res 202:2702–2719. 10.1007/s12011-023-03864-0 37770673 10.1007/s12011-023-03864-0

[21] Cengiz M, Gür B, Gür F, Şahintürk V, Bayrakdar A, Şahin IK et al (2024) The protective effects of selenium and boron on cyclophosphamide-induced hepatic oxidative stress, inflammation, and apoptosis in rats. Heliyon 10:e38713. 10.1016/j.heliyon.2024.e38713 39416834 10.1016/j.heliyon.2024.e38713 PMC11481652

[22] Onur S, Cengiz M, Ayhanci A (2024) The protective effects of boric acid against acrylamide-induced pulmonary damage in rats. 10.29261/pakvetj/2024.229

[23] Costello RB, Leser M, Coates PM (2009) Dietary supplements: current knowledge and future frontiers. In: Bales C, Ritchie C (eds) Handbook clinical nutrition aging: nutrition and health. Humana Press, Totowa, NJ. 10.1007/978-1-60327-385-5_28

[24] Ince S, Keles H, Erdogan M, Hazman O, Kucukkurt IJD (2012) Protective effect of boric acid against carbon tetrachloride–induced hepatotoxicity in mice. Drug Chem Toxicol 35:285–92. 10.3109/01480545.2011.607825 21999471 10.3109/01480545.2011.607825

[25] Donmez DB, Kacar S, Bagci R, Sahinturk V (2020) Protective effect of carnosic acid on acrylamide-induced liver toxicity in rats: mechanistic approach over Nrf2-Keap1 pathway. J Biochem Mol Toxicol 34:e22524. 10.1002/jbt.22524 32383547 10.1002/jbt.22524

[26] Li S-x, Cui N, Zhang C-l, Zhao X-l, Yu S-f, Xie K (2006) Effect of subchronic exposure to acrylamide induced on the expression of bcl-2, bax and caspase-3 in the rat nervous system. Toxicology 217:46–53. 10.1016/j.tox.2005.08.018 16242231 10.1016/j.tox.2005.08.018

